# Anti-obesity medications and cognitive disorder risk: a discrepancy between RCTs and real-world evidence

**DOI:** 10.3389/fphar.2026.1859388

**Published:** 2026-06-30

**Authors:** Chengwen Li, Chu Lin, Zonglin Li, Fang Lv, Wenjia Yang, Linong Ji, Xiaoling Cai

**Affiliations:** 1 Department of Endocrinology and Metabolism, Peking University People’s Hospital, Beijing, China; 2 Peking University Diabetes Center, Beijing, China; 3 Beijing Key Laboratory of Innovative Drug and Device Translation in Endocrine and Metabolic Diseases, Beijing, China

**Keywords:** alzheimer’s disease, anti-obesity medications, cognitive disorder, dementia, meta-analysis

## Abstract

**Objectives:**

To investigate the association between anti-obesity medication (AOM) use and the risk of cognitive disorder in individuals with overweight and obesity.

**Methods:**

We systematically searched PubMed, Embase, the Cochrane Central Register of Controlled Trials, Web of Science and Clinicaltrials.gov from inception to August 2025 for randomized controlled trials (RCTs) and observational studies of AOMs. Relative risks were calculated using a random-effect model.

**Results:**

Our analysis included 4 RCTs (n = 35,924; 17,963 AOM users, 17,961 placebo recipients) and 2 retrospective cohort studies (n = 2,303,492; 1,151,746 GLP-1RA users, 1,151,746 non-users). In RCTs, the use of AOMs was not associated with a lower risk of cognitive disorder (RR = 0.94, 95% CI 0.39 to 2.25, P = 0.88) or dementia (RR = 1.02, 95% CI 0.41 to 2.54, P = 0.97) versus placebo. Every 5-kg weight reduction mediated by AOMs was not associated with a decreased risk of cognitive disorder (RR = 0.95, 95% CI 0.44 to 2.05, P = 0.82) compared with placebo. Meta-regression further confirmed that neither absolute weight loss nor weight reduction difference between AOM and placebo groups was associated with a reduced risk of cognitive disorder. However, retrospective cohort studies showed that GLP-1RA users had lower risks of cognitive disorder (OR = 0.44, 95% CI 0.21 to 0.91, P = 0.03) and Alzheimer’s disease (OR = 0.43, 95% CI 0.19 to 0.97, P = 0.04) than non-users.

**Conclusion:**

While short-term RCTs did not observe a substantial cognitive benefit of AOMs, real-world evidence indicated that GLP-1RAs may offer potential protection against cognitive disorders. These divergent findings highlight the need for long-term prospective trials with dedicated cognitive endpoints.

## 1 Introduction

Obesity has become a major global public health challenge, affecting more than 650 million adults worldwide and contributing to the burden of numerous chronic diseases, including cognitive decline and dementia (Abad-Jiménez and Vezza, 2025; Dye et al., 2017). The complex relationship between obesity and cognitive impairment is increasingly recognized, with mounting evidence suggesting that obesity may accelerate cognitive deterioration and raise the risk of dementia through multiple biological pathways (Henn et al., 2022; Buie et al., 2019; Koriath and Perneczky, 2025; Willette et al., 2015; Markus and Joutel, 2025).

Excess adipose tissue triggers chronic low-grade systemic inflammation, which creates a pro-inflammatory environment that may promote neurodegeneration (Buie et al., 2019; Koriath and Perneczky, 2025). Obesity is also accompanied by metabolic disturbances—including insulin resistance and hyperinsulinemia—that impair cerebral glucose metabolism and facilitate the accumulation of amyloid-beta, a pathological hallmark of Alzheimer’s disease (Buie et al., 2019; Willette et al., 2015). In addition, obesity-related vascular complications may lead to cerebrovascular damage, further compromising cognitive function (Buie et al., 2019; Markus and Joutel, 2025).

Given the well-documented link between obesity and cognitive decline, weight loss holds therapeutic potential to reduce neurocognitive risk. Theoretically, weight reduction through lifestyle modifications or pharmacological interventions could mitigate cognitive risk in individuals with overweight and obesity by reversing obesity-associated pathological process (Veronese et al., 2017). Previous studies have reported reduced risks of cognitive decline or dementia among users of anti-obesity medications (AOMs) (Wang et al., 2025; Lin et al., 2025; Cheng et al., 2025; Akimoto et al., 2020), implying possible cognitive benefits from pharmacologically induced weight loss. However, most of these findings have been reported in patients with type 2 diabetes mellitus (T2DM), and the specific cognitive effects of AOMs in non-diabetic individuals with overweight and obesity remain unclear.

This uncertainty is particularly relevant for glucagon-like peptide-1 receptor agonists (GLP-1RAs) and other types of AOMs, as their cognitive impacts in the general population with overweight and obesity have not been comprehensively evaluated. Against this background, we performed an up-to-date systematic review and meta-analysis to clarify the association between anti-obesity medication use and the risk of cognitive disorder (including mild cognitive impairment and dementia) in individuals with overweight and obesity. By synthesizing data from both RCTs and real-world observational studies, we aimed to generate evidence and provide valuable insights for clinical decision-making regarding anti-obesity medications in obesity management.

## 2 Methods

### 2.1 Study design and registration

This systematic review and meta-analysis was performed in accordance with the recommendations of the Preferred Reporting Items for Systematic Reviews and Meta-analyses (PRISMA) protocol. It has been registered in the International Prospective Register of Systematic Reviews (PROSPERO) under registration number CRD420251129527.

### 2.2 Data sources and search strategy

Two independent investigators conducted comprehensive literature searches in PubMed, Embase, Web of Science, the Cochrane Central Register of Controlled Trials, and ClinicalTrials.gov to identify relevant studies investigating GLP-1RAs (mono-, dual-, and triple-agonists) and other types of AOMs (cagrilintide, phentermine plus topiramate, and naltrexone plus bupropion). The search covered all available records from each database’s inception to August 2025. The search strategy followed the Cochrane Handbook for Systematic Reviews of Interventions, utilizing both medical subject headings (MeSH) and free-text keywords. Key search terms encompassed glucagon-like peptide-1 receptor agonists, individual drug names (liraglutide, semaglutide, benaglutide, orforglipron, tirzepatide, mazdutide, cotadutide, survodutide, maritide, retatrutide, cagrilintide, phentermine plus topiramate and naltrexone plus bupropion), study design (randomized controlled trial), comparator (placebo), and condition (overweight/obesity). The full search strategy was summarized in Supplementary Table S1.

### 2.3 Study selection criteria

The inclusion criteria for this meta-analysis were as follows: (1) randomized placebo-controlled trials or observational studies investigating anti-obesity medications; (2) studies reporting cognitive disorder events (a composite endpoint consisting of mild cognitive impairment and dementia, including Alzheimer’s disease and vascular dementia); (3) publications in English. The exclusion criteria were as follows: (1) studies involving pediatric patients; (2) studies not conducted in patients with overweight or obesity; (3) studies using drugs not approved for weight loss or doses of approved drugs outside their licensed weight-loss indication; (4) studies comparing anti-obesity medications with other active drugs.

### 2.4 Data extraction and quality evaluation

According to the inclusion and exclusion criteria above, two investigators (Chengwen Li and Chu Lin) independently conducted the screening process by reviewing titles, abstracts, and full texts, removing duplicates and ineligible studies. They assessed the quality and risk of bias for each article and performed data extraction from eligible studies. The data they extracted encompassed first author, publication year, study design, follow-up duration, drug exposure, sample sizes in both experimental and control groups, patient characteristics (mean age, male percentage, body mass index [BMI], baseline weight, weight change), and cognitive disorder events (a composite endpoint consisting of mild cognitive impairment and dementia, including Alzheimer’s disease and vascular dementia.). The pre-specific dementia events included in this meta-analysis were Alzheimer’s disease, vascular dementia, frontotemporal dementia, and dementia with Lewy bodies. If relevant data were unavailable in the original articles or their Supplementary Material, we retrieved such data from Clinicaltrials.gov using the unique registered NCT trial number. All extracted data were cross-checked. Any discrepancies about data extraction were resolved by discussion with a third investigator (Xiaoling Cai). If disagreement persisted, a fourth investigator adjudicated.

### 2.5 Risks of bias assessment

The Newcastle-Ottawa Scale (NOS) was used to evaluate the risk of bias in observational studies, assessing three domains including selection of study groups, comparability of groups, and ascertainment of exposure or outcome. The total NOS scores range from 0 to 9. The Cochrane Risk of Bias tool (RoB 2.0) was employed to assess the methodological quality of included randomized controlled trials. This evaluation systematically examined five core domains: (1) randomization process, (2) deviations from intended interventions, (3) missing outcome data, (4) outcome measurement, and (5) selective reporting. Each domain was independently evaluated and classified following the tool’s standardized criteria, resulting in one of three predefined risk categories: ‘low risk’, ‘some concerns’, or ‘high risk’. Publication bias was assessed with funnel plots and Egger’s test.

### 2.6 Data synthesis and statistical analysis

The primary endpoint of this meta-analysis was the association between treatment with anti-obesity medication and the risk of cognitive disorder events. The reported dementia and different subtypes of dementia, including Alzheimer’s disease, vascular dementia, frontotemporal dementia, and dementia with Lewy bodies were considered as the exploratory endpoints.

The means and standard deviations (SDs) of weight changes were collected following the use of AOMs for each included study. The weighted mean difference (WMD) and 95% confidence interval (CI) for weight change from baseline were computed using Stata software (version 18.0; StataCorp) and Review Manager software (version 5.3; Nordic Cochrane Centre, Copenhagen, Denmark). Effect sizes were presented as risk ratio (RR) or odds ratio (OR) with 95% confidence interval (CI), computed through the Mantel-Haenszel random-effects model to address between-study heterogeneity. Study heterogeneity was assessed using the Higgins I2 statistic, with values > 50% indicating substantial heterogeneity. For all statistical tests, a two-tailed P-value < 0.05 was considered statistical significance.

Subgroup analysis was stratified by baseline characteristics and treatment-related factors, including diabetes status (with or without T2DM) and drug type (GLP-1RAs or other AOMs), to evaluate their potential confounding effects on study outcomes. Meta-regression analysis assessed the influence of absolute weight change and weight change difference on the risk of dementia in patients receiving anti-obesity medication therapies.

All statistical analyses were performed using Review Manager (Version 5.3; Nordic Cochrane Center, Copenhagen, Denmark) and STATA 15.0 (STATA Corp, College Station, TX, United States of America). The quality of evidence was assessed with the Grading of Recommendations, Assessment, Development and Evaluation (GRADE) approach.

## 3 Results

### 3.1 Baseline characteristics and quality assessment of included studies

**FIGURE 1:**
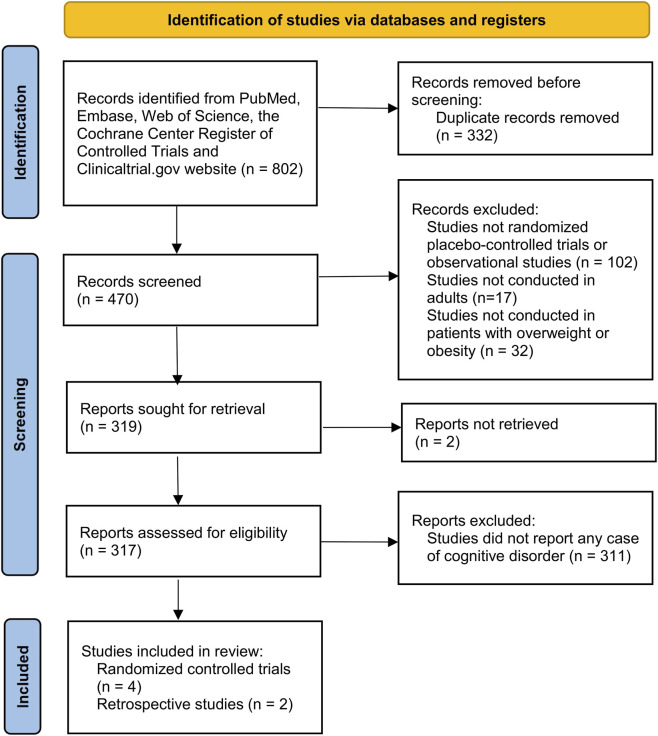
PRISMA flow diagram of included studies.

As shown in Figure 1, four randomized controlled trials (RCTs) and two retrospective cohort studies were included. The RCTs involved 17,963 participants in the experimental group and 17,961 participants in the placebo-controlled group. The cohort studies included 1,151,746 participants in the GLP-1RA group and 1,151,746 participants in the non-GLP-1RA group. There were four studies investigating GLP-1RAs, one investigating naltrexone/bupropion, and one investigating bimagrumab. The mean age of all participants was 54.9 ± 13.0 years old; the mean male percentage was 41.9%; the mean BMI was 33.9 ± 5.5 kg/m2; the mean body weight at baseline was 97.7 ± 18.9 kg and the median follow-up duration was 110 weeks. Detailed baseline characteristics of the included studies were systematically summarized in Supplementary Table S2.

The quality of observational studies and randomized controlled trials (RCTs) was assessed using the Newcastle-Ottawa Scale (NOS) and the Cochrane Risk of Bias 2.0 (RoB 2) tool, respectively. The results were presented in Supplementary Table S3, Supplementary Table S4 and Supplementary Figure S1, indicating a generally low risk of bias across the included studies. The average NOS score for observational studies was 7.5, with all of them classified as high quality (score ≥ 7/9). All four included RCTs demonstrated a low risk of bias in domains D1 (Bias arising from the randomization process), D2 (Bias due to deviations from intended interventions), D4 (Bias in measurement of the outcome), and D5 (Bias in selection of the reported result). Only one trial (Heymsfield et al., 2021) raised some concerns about bias related to missing outcome data (Domain D3).

Publication bias was assessed with funnel plots and Egger’s test. The funnel plots exhibited generally even distribution (Supplementary Figures S4, 5), while Egger’s test suggested potential publication bias for cognitive disorder outcomes in the observational studies (β = 0.953, P = 0.023; Supplementary Table S5), but not for other endpoints. However, given the small number of observational studies (n = 2), Egger’s test is inherently unstable, so this result should be interpreted with caution.

### 3.2 The association between the use of AOMs and the risk of cognitive disorder

**FIGURE 2:**
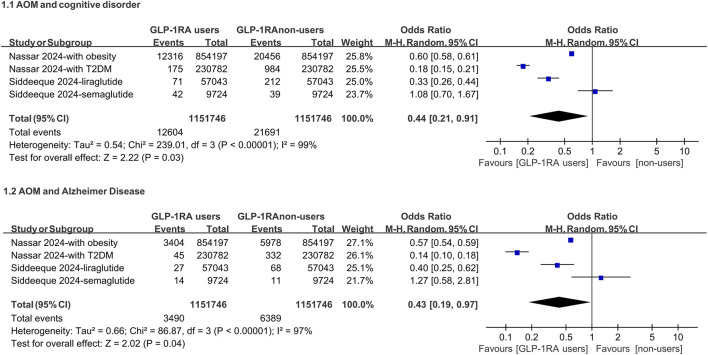
This figure illustrates the association between anti-obesity medication treatments and the risk of predefined cognitive disorder in included retrospective cohort studies of patients with overweight and obesity, showing the odds ratio (OR) and 95% confidence intervals (CI) for: overall cognitive disorder, and Alzheimer’s Disease. AOM, anti-obesity medication; T2DM, type 2 diabetes mellitus.

Meta-analysis of included retrospective cohort studies revealed that GLP-1RAs use was associated with a reduced risk of cognitive disorder (OR = 0.44, 95% CI 0.21 to 0.91, P = 0.03) and Alzheimer’s disease (OR = 0.43, 95% CI 0.19 to 0.97, P = 0.04) compared with the non-GLP-1RA group (Figure 2).

**FIGURE 3:**
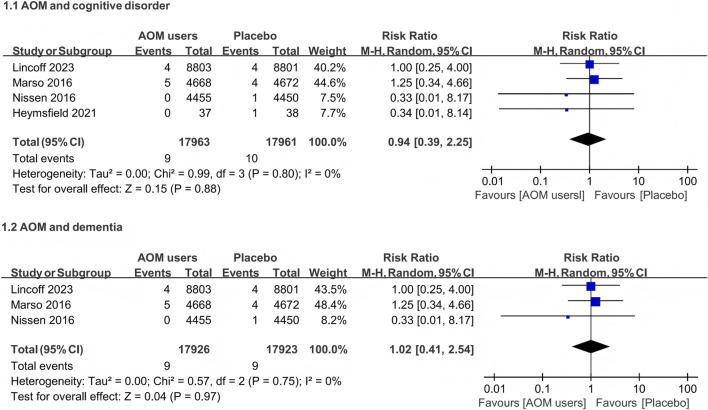
This figure illustrates the association between anti-obesity medication treatments and the risk of cognitive disorder in included randomized controlled studies of patients with overweight and obesity, showing the risk ratio (RR) and 95% confidence intervals (CI) for: overall cognitive disorder, and dementia. AOM, anti-obesity medication.

In contrast, the meta-analysis of included RCTs found no association between the use of AOMs and reduced risk of cognitive disorder (RR = 0.94, 95% CI 0.39 to 2.25, P = 0.88) or dementia (RR = 1.02, 95% CI 0.41 to 2.54, P = 0.97) compared with placebo (Figure 3).

**FIGURE 4:**
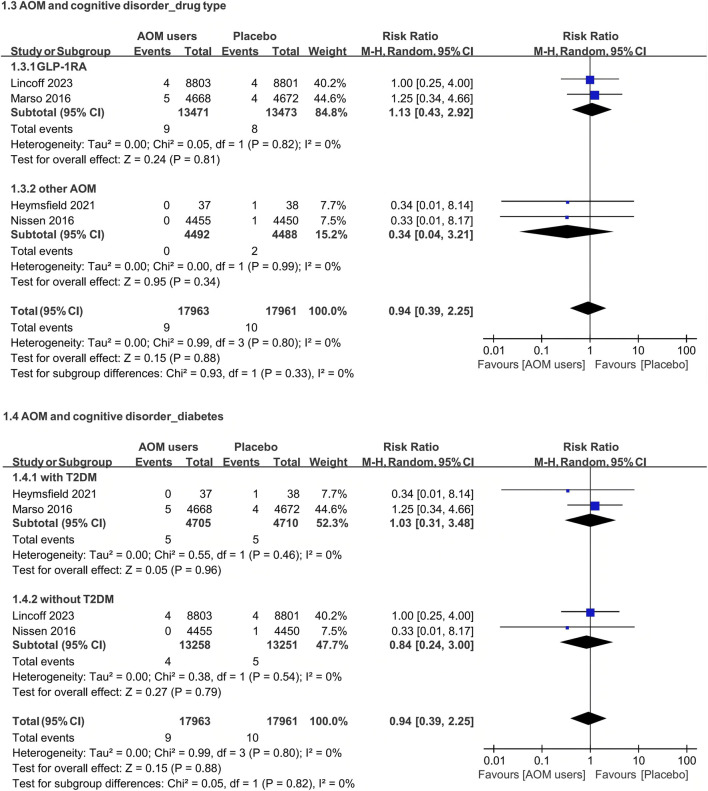
This figure illustrates the subgroup analysis of the association between anti-obesity medication treatments and the risk of cognitive disorder stratified by drug type and diabetes status in included randomized controlled studies of patients with overweight and obesity, showing the risk ratio (RR) and 95% confidence intervals (CI). AOM, anti-obesity medication; T2DM, type 2 diabetes mellitus.

In subgroup analysis by drug type, no association was observed between the use of GLP-1RAs and the risk of cognitive disorder (RR = 1.13, 95% CI 0.43 to 2.92, P = 0.81) compared with placebo. Similarly, the use of other AOMs, including naltrexone/bupropion and bimagrumab, was not associated with risk reduction of cognitive disorder (RR = 0.33, 95% CI 0.04 to 3.17, P = 0.34) compared with placebo (Figure 4).

### 3.3 The effect of weight loss mediated by AOMs on the risk of cognitive disorder

When compared with placebo, each 5-kg AOM-mediated weight reduction was not associated with a decreased risk of cognitive disorder (RR = 0.95, 95% CI 0.44 to 2.05, P = 0.82; Supplementary Figure S2). Meta-regression analysis further revealed that neither absolute weight loss mediated by AOMs (β = 0.80, 95% CI, -5.44 to 7.03, p = 0.74) nor the weight reduction difference between AOM and placebo groups (β = 0.75, 95% CI, -4.11 to 5.60, P = 0.69) was associated with a reduced risk of cognitive disorder (Supplementary Figure S3).

## 4 Discussion

Our meta-analysis revealed a striking divergence between RCTs and real-world evidence regarding AOMs and cognitive disorders. In short-term RCTs, the use of anti-obesity medications was not associated with a reduced risk of cognitive disorder in patients with overweight or obesity, while retrospective cohort studies suggested that the use of GLP-1RA was associated with a reduced risk of cognitive disorder and Alzheimer’s disease compared with the non-GLP-1RA group. Several key factors may explain these conflicting results.

First, the included RCTs were primarily designed and powered to assess cardiovascular and metabolic outcomes, with cognitive disorders documented only as adverse events rather than prespecified primary endpoints (Lincoff et al., 2023; Heymsfield et al., 2021; Nissen et al., 2016; Marso et al., 2016). Furthermore, the median 110-week follow-up in these trials was too short to detect slowly progressive cognitive disorders, which typically develop over years. In contrast, real-world observational studies had longer follow-up periods, enabling more reliable detection of long-term cognitive effects of AOMs.

Second, the observed risk reduction in cohort studies could be influenced by unmeasured confounding factors. In clinical practice, patients prescribed GLP-1RAs often adopt healthier lifestyles, engage more consistently with medical care, and have more favorable socioeconomic profiles compared to non-users. These factors are independently associated with a lower risk of cognitive decline and may partially explain the observed protective effect. By contrast, randomization in RCTs effectively balances such prognostic factors across groups, thereby minimizing this type of confounding.

Third, heterogeneity in drug exposure represented another key consideration. Randomized controlled trials assess the effects of a fixed, standardized drug regimen under tightly controlled conditions over a predefined treatment period. By contrast, observational studies tend to include patients with longer cumulative exposure to GLP-1RAs and higher rates of long-term treatment adherence, which could translate into a more pronounced cumulative neurocognitive effect.

Our subgroup and meta-regression analyses were performed to identify potential effect modifiers. Subgroup analysis stratified by drug type showed that the use of GLP-1RAs alone was not associated with a reduced risk of cognitive disorder in RCTs. Likewise, the use of other AOMs, including naltrexone/bupropion and bimagrumab, also yielded null associations. Furthermore, we found no association between the degree of weight loss and cognitive risk reduction. In particular, each 5-kg weight reduction mediated by AOMs was not associated with a decreased risk of cognitive disorder. Meta-regression analysis confirmed this, revealing that neither absolute weight loss mediated by AOMs nor the weight reduction difference between AOM and placebo groups was associated with the relative risk of cognitive disorder.

Notably, our RCT meta-analysis found no association between AOM-mediated weight loss and reduced cognitive disorder risk. Existing literature suggests that GLP-1RAs may exert neuroprotective effects through weight-independent pathways. Specifically, direct activation of GLP-1 receptors in the hippocampus can enhance synaptic plasticity and promote neuronal survival. Furthermore, GLP-1RAs have been shown to attenuate microglial activation and neuroinflammation, as well as reduce amyloid-beta accumulation and tau phosphorylation (Li et al., 2010; McClean et al., 2011; Zhang et al., 2025; Roy et al., 2025), which are key pathological features of Alzheimer’s disease. These direct central nervous system (CNS) effects contrast with other AOMs like naltrexone/bupropion. While naltrexone/bupropion targets the hypothalamic pro-opiomelanocortin (POMC) system and inhibites dopamine/norepinephrine reuptake (Sherman et al., 2016), it lacks the neurotrophic and anti-inflammatory receptor profiles of GLP-1RAs. Unlike GLP-1RAs, it does not directly activate synaptic plasticity pathways (e.g., PI3K/Akt) or mitigate neuroinflammation via microglial modulation, which may explain their divergent cognitive outcomes. Additionally, the observed real-world cognitive benefits might partly reflect GLP-1RA-mediated improvements in vascular risk factors. For instance, GLP-1RAs can reduce systemic blood pressure and attenuate endothelial dysfunction by upregulating endothelial nitric oxide synthase (eNOS) activity (Oh et al., 2026; Helmstädter et al., 2020). These vascular benefits are particularly relevant for mitigating vascular dementia risk. Accordingly, the cognitive effects of AOMs should be interpreted with careful attention to both weight-dependent and weight-independent mechanisms. Future studies should be explicitly designed to disentangle these distinct pathways.

Limitations of our study should be acknowledged. First, the number of included studies was relatively limited. Only two RCTs in our analysis investigated GLP-1RAs, which provided insufficient statistical power to perform detailed subgroup analyses. For instance, we were unable to compare short-acting agents (e.g., liraglutide) with long-acting ones (e.g., semaglutide), or mono-agonists with dual/triple agonists (e.g., tirzepatide, retatrutide). As for GLP-1RA, different types of them may exert distinct effects on cognitive function, but the available data from the included studies is insufficient to perform a subgroup analysis to directly compare the cognitive effects of specific GLP-1RA against each other.

Second, the majority of evidence came from observational studies, which are inherently susceptible to residual confounding relevant to cognitive risk. Despite adjustments in the original studies, unmeasured or imperfectly measured factors—such as genetic susceptibility, lifestyle factors, and concurrent comorbidities—could have potentially biased the observed associations.

Third, most of the protective effect in the real-world evidence originated from diabetic populations; therefore, caution is warranted when extrapolating these potential cognitive benefits to non-diabetic individuals with isolated obesity. Furthermore, our analysis could not account for prediabetes status as a distinct category due to lack of stratified data in the included studies, which represents an important gap given the heightened metabolic and cognitive risks in this transitional state.

Fourth, the anti-obesity medications (AOMs) evaluated in this meta-analysis were restricted to GLP-1RAs (liraglutide and semaglutide) and a few non-GLP-1 agents (naltrexone/bupropion and bimagrumab). Our findings may not be generalizable to other types of AOMs such as orlistat or phentermine/topiramate.

Finally, we were unable to directly compare the cognitive effects of AOMs with those of established interventions such as bariatric surgery or intensive lifestyle modification. Given that these alternative approaches also demonstrate metabolic benefits and may influence cognitive health through distinct or overlapping pathways, the relative efficacy and risk-benefit profiles of pharmacological versus non-pharmacological strategies for obesity management remain an important unanswered question.

Future research should address these limitations by: (1) extending follow-up periods and enrolling larger samples to enable assessment of long-term cognitive outcomes; (2) incorporating a broader range of weight-loss interventions—including lifestyle modifications and bariatric surgery—for head-to-head comparisons; (3) collecting comprehensive baseline data on risk factors and comorbidities to enhance confounding adjustment; (4) directly comparing cognitive effects between different GLP-1RA types (e.g., liraglutide vs. Semaglutide, mono-agonists vs. dual/triple agonists) and other AOM types; (5) extending investigations to non-diabetic populations with obesity to verify whether the potential cognitive benefits observed in diabetic cohorts can be generalized; and (6) further exploring the mechanistic basis of GLP-1RAs and other AOMs to support personalized therapeutic strategies for patients with obesity and high cognitive risk.

## 5 Conclusion

In conclusion, our meta-analysis revealed a complex and nuanced pattern. Although the anti-obesity medications evaluated in RCTs (including liraglutide, semaglutide, naltrexone/bupropion, and bimagrumab) showed no cognitive risk-lowering effect, real-world evidence pointed to a potential neuroprotective role of GLP-1RAs. These findings highlight the need for long-term prospective clinical trials with cognitive function as a prespecified primary endpoint and support considering the potential neurological benefits of AOMs in clinical decision-making for obesity management, especially in individuals at high risk of dementia.

## Data Availability

The original contributions presented in the study are included in the article/Supplementary Material, further inquiries can be directed to the corresponding authors.
