# Supplementary material for: Anti-obesity medications and cognitive disorder risk: a discrepancy between RCTs and real-world evidence
Source: Front Pharmacol. 2026 Jun 30;17:1859388. doi: 10.3389/fphar.2026.1859388 (PMC13365254; doi:10.3389/fphar.2026.1859388)
Supplement: Supplementary file 1 [file DataSheet1.PDF]

## *Supplementary Material*

**Supplementary Tables**

Table S1. Search strategy used for Pubmed, Medline, Embase, Web of Science, the Cochrane Center Register of Controlled Trials and Clinicaltrial.gov database

Table S2. Baseline characteristics of studies included in this meta-analysis

Table S3. Risk of bias in included observational studies (by Newcastle-Ottawa Scale)

Table S4. Risk of bias in included randomized controlled trials (by Cochrane RoB 2 tool)

Table S5. Egger's test for research endpoints

**Supplementary Figures**

Figure S1. Risk of bias summary for included randomized controlled trials (RCTs)

Figure S2. Standardized effect of every 5kg body weight change on risk of cognitive disorder

Figure S3. Meta-regression analysis for absolute weight change and weight change difference between AOMs/placebo groups and the risk of cognitive disorder in patients with overweight and obesity

Figure S4. Funnel plots of analysis endpoints in observational studies

Figure S5. Funnel plots of analysis endpoints in randomized controlled trials

**Table S1. Search strategy used for Pubmed, Medline, Embase, the Cochrane Center Register of Controlled Trials, Web of Science and Clinicaltrial.gov database.**

**Search strategy (PubMed/MEDLINE database)**

**Population:**

**#1** ("Overweight"[MeSH Terms] OR "Obesity"[MeSH Terms] OR "Obese"[Title/Abstract])

**Interventions:**

**#2** ("Glucagon-Like Peptide-1 Receptor Agonists"[MeSH Terms] OR "Glucagon Like Peptide 1 Receptor Agonists" [Title/Abstract] OR "GLP-1 Analogs" [Title/Abstract] OR "GLP 1 Analogs" [Title/Abstract] OR "GLP-1 Receptor Agonists" [Title/Abstract] OR "GLP 1 Receptor Agonists" [Title/Abstract] OR "Liraglutide" [Title/Abstract] OR "Semaglutide" [Title/Abstract] OR "Danuglipron" [Title/Abstract] OR "Orforglipron" [Title/Abstract] OR "Benaglutide" [Title/Abstract] OR "Tirzepatide"[Title/Abstract] OR "Cotadutide"[Title/Abstract] OR "Mazdutide"[Title/Abstract] OR "Survodutide"[Title/Abstract] OR "Cagrilintide"[Title/Abstract] OR "Maritide"[Title/Abstract] OR "Bimagrumab"[Title/Abstract] OR "Retatrutide"[Title/Abstract] OR "Phentermine plus topiramate"[Title/Abstract] OR "Phentermine-topiramate"[Title/Abstract] OR "Phentermine/topiramate"[Title/Abstract] OR "Naltrexone plus bupropion"[Title/Abstract] OR "Naltrexone-bupropion"[Title/Abstract] OR "Naltrexone/bupropion"[Title/Abstract])

**Study design:**

**#3** ("Randomized controlled trial"[Publication Type])

**#4** ("Placebos"[Title/Abstract] OR "Placebo"[Title/Abstract] OR "Placebo-controlled"[Title/Abstract])

**Final search strategies:**

**#1 AND #2 AND #3 AND #4**

**Search strategy (EMBASE database)**

**Population:**

**#1** "Overweight"/exp OR "Obesity"/exp OR "Obese":ti,ab

**Interventions:**

**#2** "Glucagon-Like Peptide-1 Receptor Agonist"/exp OR "Glucagon Like Peptide 1 Receptor Agonists":ti,ab OR "GLP-1 Analogs":ti,ab OR "GLP 1 Analogs":ti,ab OR "GLP-1 Receptor Agonists":ti,ab OR "GLP 1 Receptor Agonists":ti,ab OR "Liraglutide"/exp OR "Semaglutide"/exp OR "Danuglipron"/exp OR "Orforglipron"/exp OR "Benaglutide":ti,ab OR "Tirzepatide"/exp OR "Cotadutide"/exp OR "Mazdutide"/exp OR "Survodutide"/exp OR "Cagrilintide"/exp OR "Maritide":ti,ab OR "Bimagrumab"/exp OR "Retatrutide"/exp OR "Phentermine plus topiramate"/exp OR "Phentermine-topiramate":ti,ab OR "Phentermine/topiramate":ti,ab OR "Naltrexone plus bupropion":ti,ab OR "Naltrexone-bupropion":ti,ab OR "Naltrexone/bupropion":ti,ab

**Study design:**

**#3** "Randomized controlled trial"/exp

**#4** "Placebos"/exp OR "Placebo":ti,ab OR "Placebo-controlled":ti,ab

**Final search strategies:**

**#1 AND #2 AND #3 AND #4 AND ('article'/it)**

**Search strategy (Web of Science database)**

**Population:**

**#1** TS=("Overweight" OR "Obesity" OR "Obese")

**Interventions:**

**#2** TS=("Glucagon Like Peptide 1 Receptor Agonists" OR "GLP-1 Analogs" OR "GLP 1 Analogs" OR "GLP-1 Receptor Agonists" OR "GLP 1 Receptor Agonists" OR "Liraglutide" OR "Semaglutide" OR "Danuglipron" OR "Orforglipron" OR "Benaglutide" OR "Tirzepatide" OR "Cotadutide" OR "Mazdutide" OR "Survodutide" OR "Cagrilintide" OR "Maritide" OR "Bimagrumab" OR "Retatrutide" OR "Phentermine plus topiramate" OR "Phentermine-topiramate" OR "Phentermine/topiramate" OR "Naltrexone plus bupropion" OR "Naltrexone-bupropion" OR "Naltrexone/bupropion")

**Study design:**

**#3** TS=("Randomized controlled trial" OR "Randomised controlled trial" OR "RCT")

**#4** TS=("Placebos" OR "Placebo" OR "Placebo-controlled")

**Final search:**

**#1 AND #2 AND #3 AND #4**

**Search strategy (the Center Register of Controlled Trials and Clinicaltrial.gov database)**

**Population:**

**#1** MeSH Terms descriptor: [Overweight] explode all trees

**#2** MeSH Terms descriptor: [Obesity] explode all trees

**#3** "Obese":ti,ab,kw

**#4 (#1 OR #2 OR #3)**

**Interventions:**

**#5** MeSH Terms descriptor: [Glucagon-Like Peptide-1 Receptor Agonist] explode all trees

**#6** ("Glucagon Like Peptide 1 Receptor Agonists" OR "GLP-1 Analogs" OR "GLP 1 Analogs" OR "GLP-1 Receptor Agonists" OR "GLP 1 Receptor Agonists" OR "Liraglutide" OR "Semaglutide" OR "Danuglipron" OR "Orforglipron" OR "Benaglutide" OR "Tirzepatide" OR "Cotadutide" OR "Mazdutide" OR "Survodutide" OR "Cagrilintide" OR "Maritide" OR "Bimagrumab" OR "Retatrutide" OR "Phentermine plus topiramate" OR "Phentermine-topiramate" OR "Phentermine/topiramate" OR "Naltrexone plus bupropion" OR "Naltrexone-bupropion" OR "Naltrexone/bupropion"):ti,ab,kw

**#7 (#5 OR #6)**

**Study design:**

**#8** ("Randomized controlled trial"):ti,ab,kw

**#9** ("Placebos" OR "Placebo" OR "Placebo-controlled"):ti,ab,kw

**Final search strategies:**

**#4 AND #7 AND #8 AND #9**

**Table S2. Baseline characteristics of studies included in this meta-analysis**

| Author, year | Follow-up duration (weeks) | Treatment group | No. of patients | Age (years) | Male (%) | BMI (kg/m$^2$) | Weight(kg) | With (1) or without (2) diabetes |
|---|---|---|---|---|---|---|---|---|
| **Retrospective cohort studies** | | | | | | | | |
| Nassar 2024-a [1] | > 24 | GLP-1RA | 854197 | 57.3 | 46.5 | / | / | 1 |
| | >24 | non-users | 854197 | 57.3 | 46.5 | / | / | 1 |
| Nassar 2024-b [1] | > 24 | GLP-1RA | 230782 | 46.5 | 25.9 | / | / | 2 |
| | >24 | non-users | 230782 | 46.5 | 25.9 | / | / | 2 |
| Siddeeque 2024-a [2] | 17.5 ± 18.4 | Semaglutide | 57043 | / | / | / | / | 1&2 |
| | 31.8 ± 31.4 | non-users | 57043 | / | / | / | / | 1&2 |
| Siddeeque 2024-b [2] | 17.5 ± 18.4 | Liraglutide | 9724 | / | / | / | / | 1&2 |
| | 31.8 ± 31.4 | non-users | 9724 | / | / | / | / | 1&2 |
| **Randomized controlled trials** | | | | | | | | |
| Lincoff, 2023 [3] | 159 | Semaglutide 2.4mgqw injection | 8803 | 61.6 | 72.2 | 33.3 | 96.5 | 2 |
| | | PBO | 8801 | 61.6 | 72.5 | 33.4 | 96.8 | 2 |

| | | | | | | | | |
|---|---|---|---|---|---|---|---|---|
| Marco, 2016 [4] | 182 | Liraglutide 1.8mgqd injection | 4668 | 64.2 | 64.5 | 32.5 | 91.9 | 1 |
| | | PBO | 4672 | 64.4 | 64 | 32.5 | 91.6 | 1 |
| Nissen, 2016 [5] | 52 | Naltrexone SR 32mg+Bupropion SR 360mg qd | 4455 | 61.1 | 45.3 | 36.6 | 105.6 | 2 |
| | | PBO | 4450 | 60.9 | 45.6 | 36.7 | 106.3 | 2 |
| Heymsfield 2021 [6] | 48 | Bimagrumab 10 mg/kg intravenous infusion | 37 | 60.7 | 37.8 | 32.7 | 90.1 | 1 |
| | | PBO | 38 | 60.2 | 68.4 | 33.1 | 96.9 | 1 |

Values are expressed as absolute number or mean±standard deviationor mean (minimal value, maximal value).

PBO: placebo; GLP-1RA: glucagon-like peptide-1 receptor agonist

**Table S3. Risk of bias in included observational studies (by Newcastle-Ottawa Scale)**

| Cohort studies | | | | | | | | | |
|---|---|---|---|---|---|---|---|---|---|
| | Selection | | | | Comparability | Outcome | | | |
| Author, year | Representativeness of the exposed cohort | Selection of the non-exposed cohort | Ascertainment of exposure | Concealed demonstration of outcomes before exposure | Comparability of cohorts on the basis of design | Assessment of outcome | Follow-up long enough for outcomes to occur | Adequacy of follow up for cohorts | Total score |
| Nassar, 2024 [1] | ★ | ★ | ★ | ★ | ★★ | ★ | ★ | ☆ | 8 |
| Siddeeque, 2017 [2] | ★ | ★ | ★ | ★ | ★★ | ★ | ☆ | ☆ | 7 |

Filled Star (★): Represents a "Yes" or a positive judgment for that specific item. It indicates that the study met the quality criterion for that domain.

Empty Star (☆): Represents a "No" or a negative judgment for that specific item. It indicates that the study did not meet the quality criterion for that domain.

The total score for a study is the sum of all the filled stars (★) across the three main domains (Selection, Comparability, and Outcome). A higher total score (maximum of 9 stars) indicates a lower risk of bias and higher methodological quality according to the NOS criteria.

**Table S4. Risk of bias in included randomized controlled trials (by Cochrane RoB 2 tool)**

| Author, year | D1 (Bias arising from the randomization process) | | | D2 (Bias due to deviations from intended interventions) | | D3 (Bias due to missing outcome data) | | D4 (Bias in measurement of the outcome) | D5 (Bias in selection of the reported result) |
|---|---|---|---|---|---|---|---|---|---|
| Lincoff, 2023 [3] | Low risk | Using an interactive voice/web response system | Generally balanced baseline characteristics across groups | Low risk | Randomized; Double blinding | Low risk | There were 2.94% (259/8803) and 3.23% (284/8801) patients in semaglutide 2.4mg and placebo groups with missing outcome data, respectively; missing outcome data were generally balanced across treatment groups, with similar reasons for missing data | Low risk | Low risk |
| Marso, 2016 [4] | Low risk | Using an interactive voice/web response system | Generally balanced baseline characteristics across groups | Low risk | Randomized; Double blinding | Low risk | There were 2.98% (139/4668) and 3.40% (159/4672) patients in liraglutide 1.8mg and placebo groups with missing outcome data, respectively; missing outcome data were generally balanced across treatment groups, with similar reasons for missing data | Low risk | Low risk |

| | | | | | | | | | |
|---|---|---|---|---|---|---|---|---|---|
| Nissen, 2016 [5] | Low risk | Using an interactive voice/web response system | Generally balanced baseline characteristics across groups | Low risk | Randomized; Double blinding | Low risk | There were 0.02% (1/4456) and 0.09% (4/4454) patients in Naltrexone SR 32mg/Bupropion SR 360mg qd and placebo groups with missing outcome data, respectively; missing outcome data were generally balanced across treatment groups, with similar reasons for missing data | Low risk | Low risk |
| Heymsfield, 2021 [6] | Low risk | Using an interactive voice/web response system | Generally balanced baseline characteristics across groups | Low risk | Randomized; Double blinding | Some concerns | There were 30.77% (12/39) and 20.51% (8/39) patients in Bimagrumab 10 mg/kg intravenous infusion and placebo groups with missing outcome data, respectively; missing outcome data were generally balanced across treatment groups | Low risk | Low risk |

**Table S5. Egger's test for research endpoints**

| Endpoints | β | 95%CI | P value |
|---|---|---|---|
| **Observational Studies** | | | |
| Cognitive disorder | 0.953 | 0.323, 1.583 | 0.023 |
| Alzheimer Disease | 1.029 | -2.321, 4.379 | 0.160 |
| **Randomized controlled trials** | | | |
| Cognitive disorder | -0.452 | -1.062, 0.158 | 0.086 |
| Dementia | -0.508 | -1.248, 0.232 | 0.098 |

**Figure S1. Risk of bias summary for included randomized controlled trials (RCTs)**

| | D1 | D2 | D3 | D4 | D5 | Overall |
|---|---|---|---|---|---|---|
| Lincoff 2023 | + | + | + | + | + | + |
| Marso 2016 | + | + | + | + | + | + |
| Nissen 2016 | + | + | + | + | + | + |
| Heymsfield 2021 | + | + | ! | + | + | ! |

Domains:

D1: Randomisation process
D2: Deviations from the intended interventions
D3: Missing outcome data
D4: Measurement of the outcome
D5: Selection of the reported result

Jugement:

+ Low risk
! Some concerns
- High risk

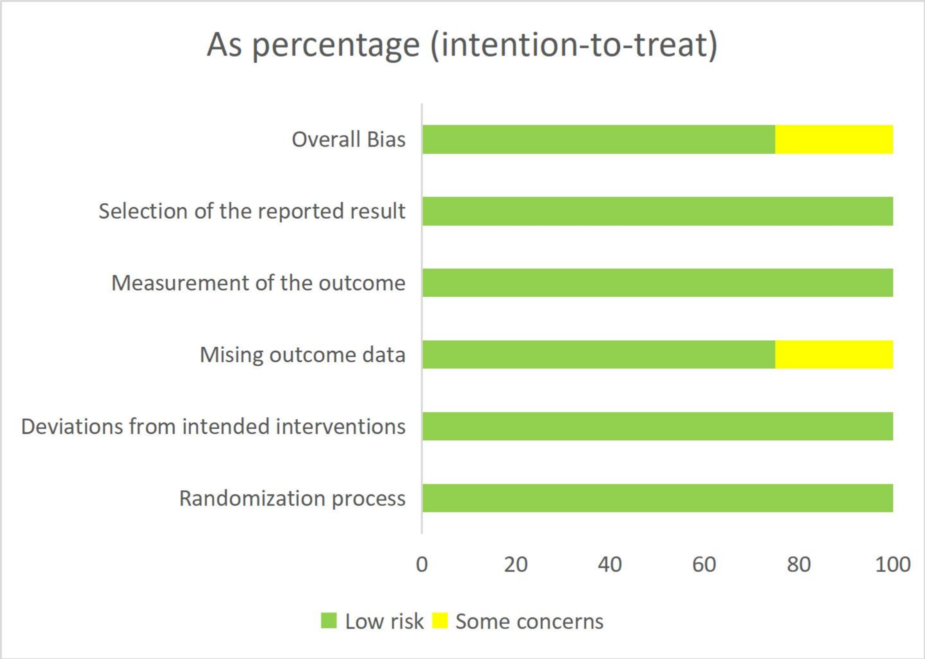

**Figure S2. Standardized effect of every 5kg body weight change on risk of cognitive disorder**

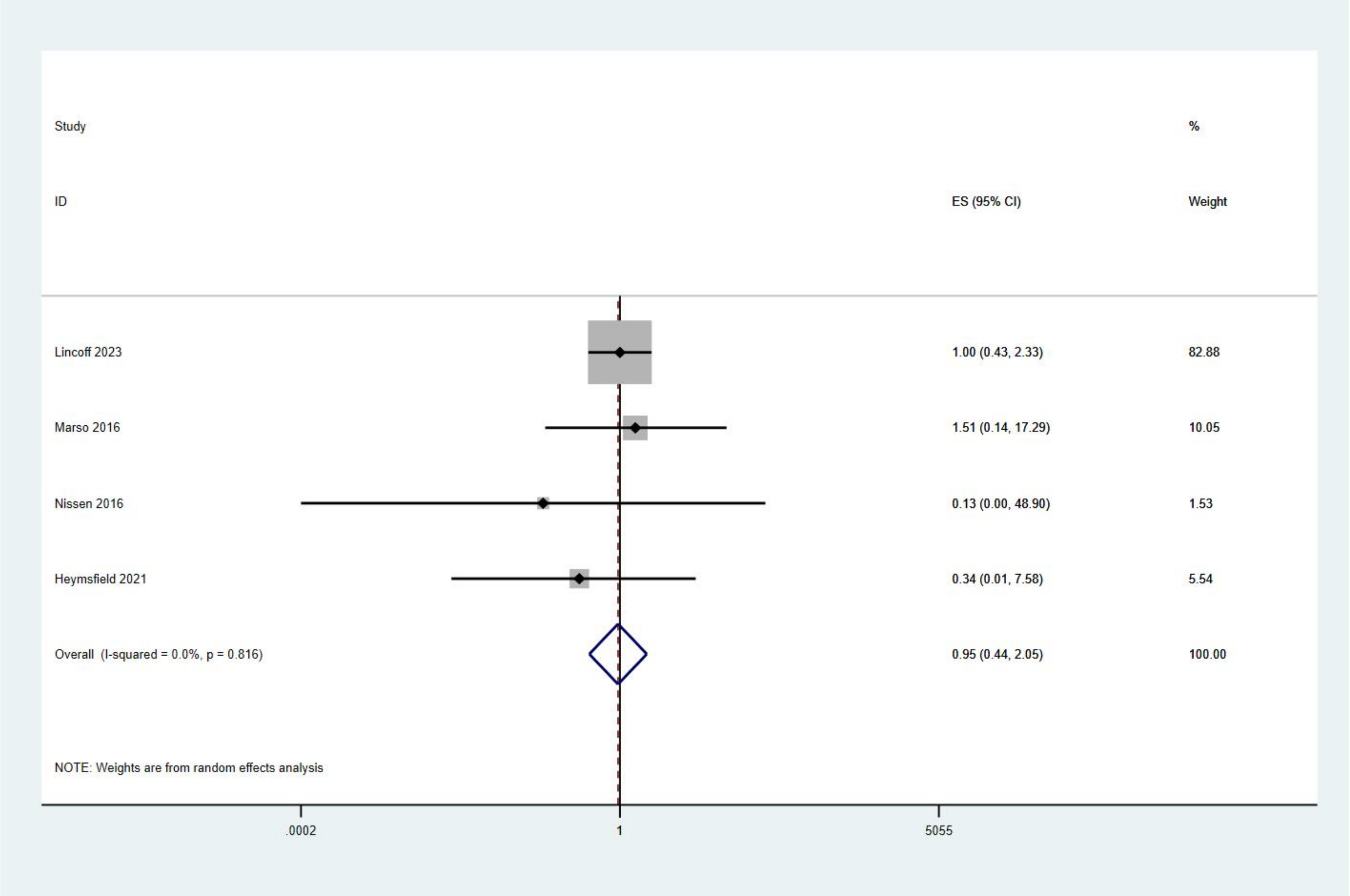

**Figure S3. Meta-regression analysis for absolute weight change and weight change difference between AOMs/placebo groups and the risk of cognitive disorder in patients with overweight and obesity**

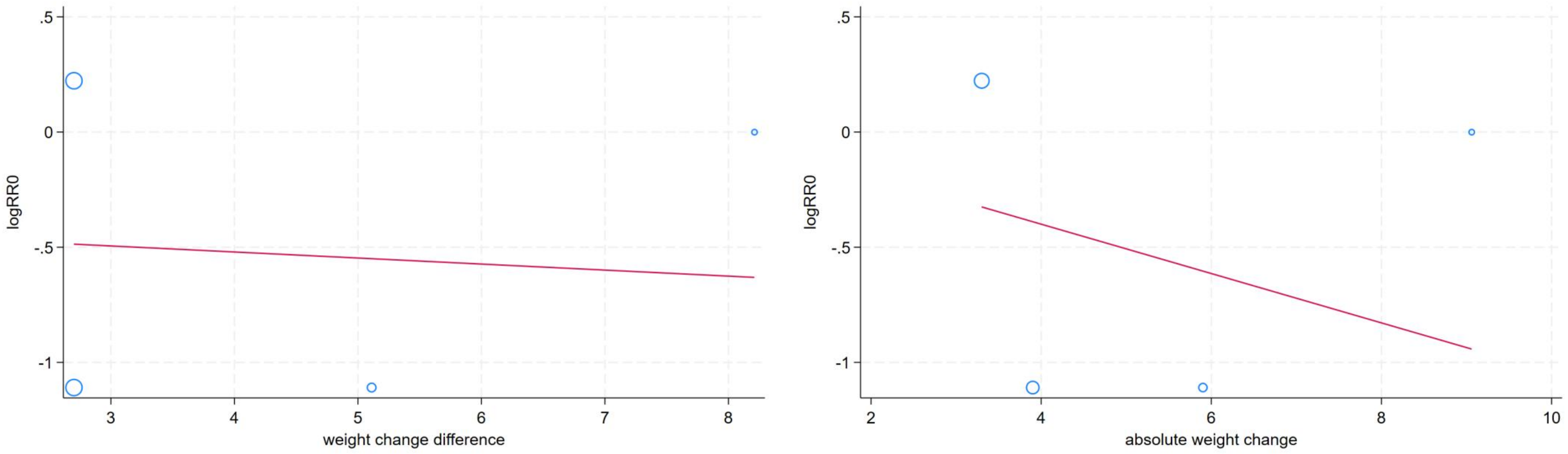


| Variables | β | 95%CI | t | p value | $I^2$ |
|---|---|---|---|---|---|
| absolute weight change | -0.42 | -7.57, 6.72 | -0.19 | 0.86 | 0% |
| weight change difference | -0.50 | -6.06, 5.06 | -0.29 | 0.79 | 0% |

**Figure S4. Funnel plots of analysis endpoints in observational studies**

- Figure S4-1. Funnel plots for the endpoints of cognitive disorder
- Figure S4-2. Funnel plots for the endpoints of Alzheimer Disease

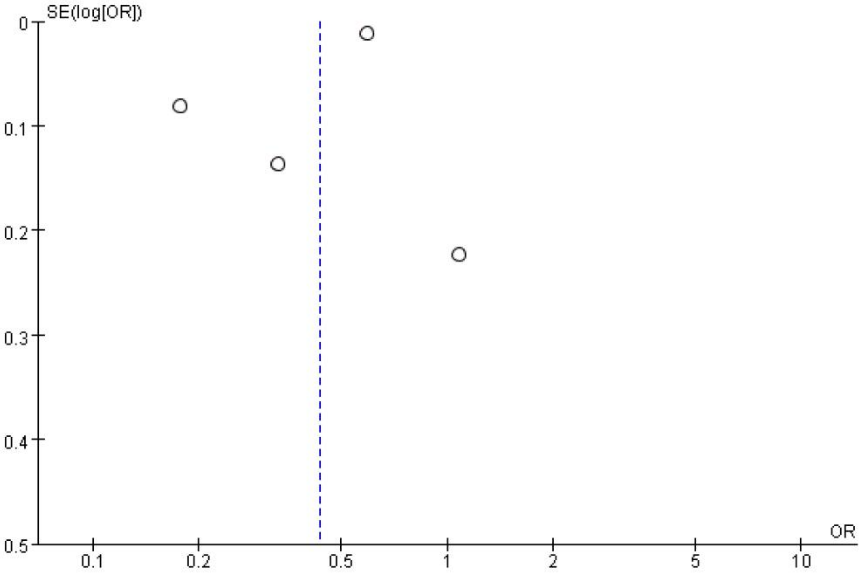


Funnel plot of comparison: AOM and cognitive disorder.

Figure S4-1

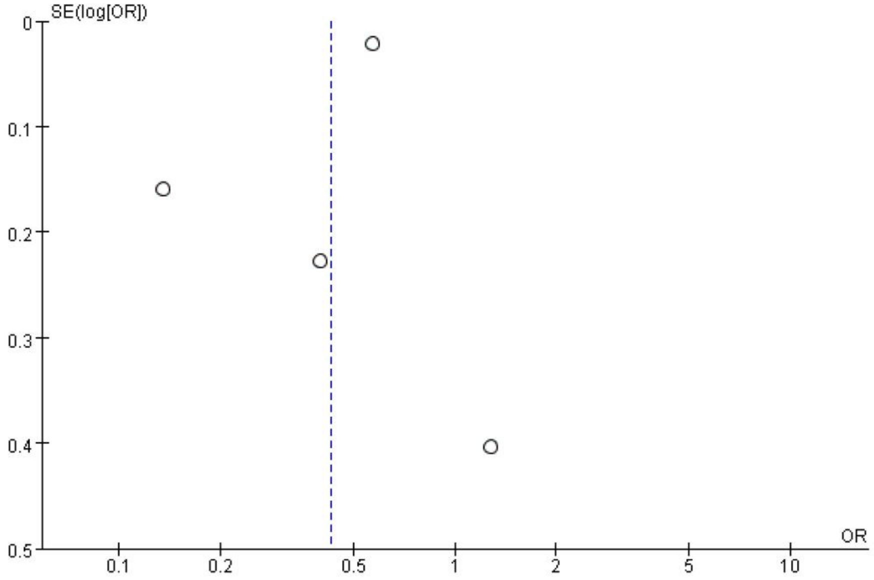


Funnel plot of comparison: AOM and Alzheimer Disease.

Figure S4-2

**Figure S5. Funnel plots of analysis endpoints in randomized controlled trials**

- Figure S5-1. Funnel plots for the endpoints of cognitive disorder
- Figure S5-2. Funnel plots for the endpoints of dementia

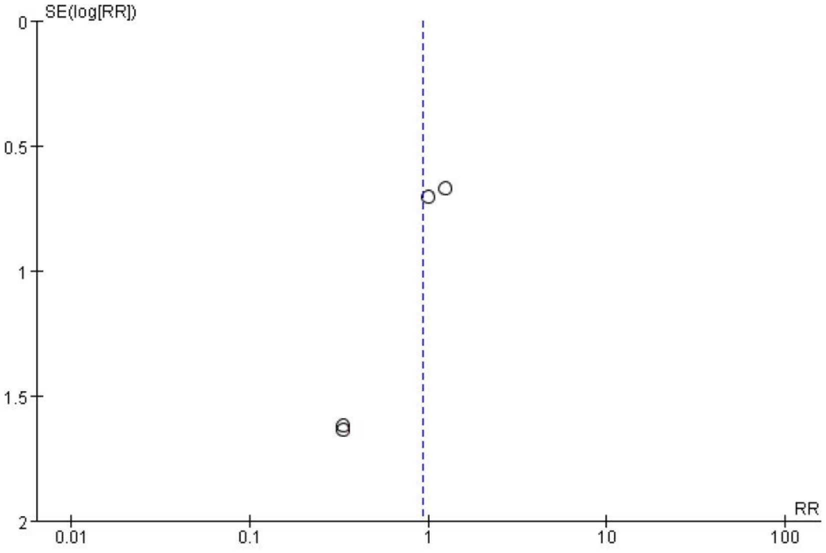


Funnel plot of comparison: AOM and cognitive disorder.

Figure S5-1

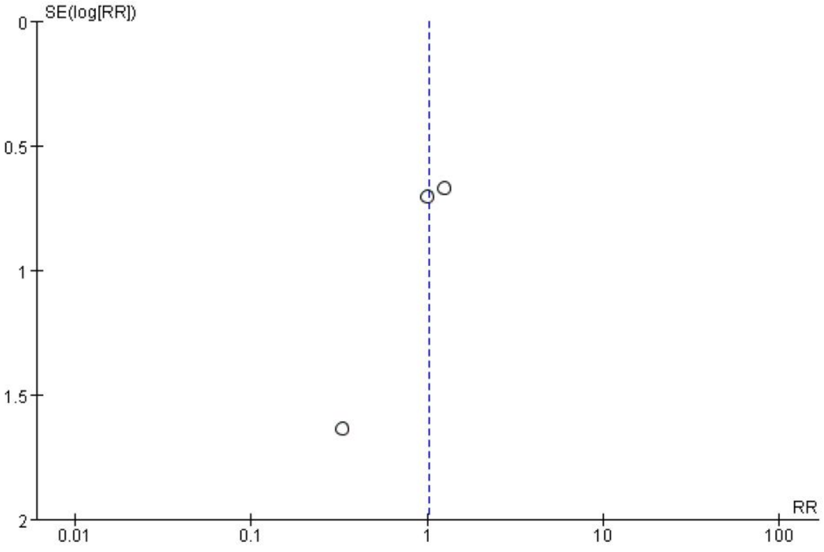


Funnel plot of comparison: AOM and dementia.

Figure S5-2

## References

[1] Nassar M, Nassar O, Abosheaishaa H, Misra A. Comparative outcomes of systemic diseases in people with type 2 diabetes, or obesity alone treated with and without GLP-1 receptor agonists: a retrospective cohort study from the Global Collaborative Network. Journal of Endocrinological Investigation. 2025/02/01 2025;48(2):483-497. doi:10.1007/s40618-024-02466-4

[2] Siddeeque N, Hussein MH, Abdelmaksoud A, et al. Neuroprotective effects of GLP-1 receptor agonists in neurodegenerative Disorders: A Large-Scale Propensity-Matched cohort study. Int Immunopharmacol. Dec 25 2024;143(Pt 3):113537. doi:10.1016/j.intimp.2024.113537

[3] Lincoff AM, Brown-Frandsen K, Colhoun HM, et al. Semaglutide and Cardiovascular Outcomes in Obesity without Diabetes. N Engl J Med. Dec 14 2023;389(24):2221-2232. doi:10.1056/NEJMoa2307563

[4] Marso SP, Daniels GH, Brown-Frandsen K, et al. Liraglutide and Cardiovascular Outcomes in Type 2 Diabetes. N Engl J Med. Jul 28 2016;375(4):311-22. doi:10.1056/NEJMoa1603827

[5] Nissen SE, Wolski KE, Prcela L, et al. Effect of Naltrexone-Bupropion on Major Adverse Cardiovascular Events in Overweight and Obese Patients With Cardiovascular Risk Factors: A Randomized Clinical Trial. Jama. Mar 8 2016;315(10):990-1004. doi:10.1001/jama.2016.1558

[6] Heymsfield SB, Coleman LA, Miller R, et al. Effect of Bimagrumab vs Placebo on Body Fat Mass Among Adults With Type 2 Diabetes and Obesity: A Phase 2 Randomized Clinical Trial. JAMA Netw Open. Jan 4 2021;4(1):e2033457. doi:10.1001/jamanetworkopen.2020.33457
